# COVID-19 in Relation to Hyperglycemia and Diabetes Mellitus

**DOI:** 10.3389/fcvm.2021.644095

**Published:** 2021-05-20

**Authors:** Hayder M. Al-kuraishy, Ali I. Al-Gareeb, M. Alblihed, Susana G. Guerreiro, Natália Cruz-Martins, Gaber El-Saber Batiha

**Affiliations:** ^1^Department of Clinical Pharmacology and Therapeutic Medicine, College of Medicine, ALmustansiriyiah University, Baghdad, Iraq; ^2^Department of Microbiology, College of Medicine, Taif University, Taif, Saudi Arabia; ^3^Faculty of Medicine, University of Porto, Porto, Portugal; ^4^Institute of Molecular Pathology and Immunology of the University of Porto (IPATIMUP), Porto, Portugal; ^5^Institute for Research and Innovation in Health (i3S), University of Porto, Porto, Portugal; ^6^Laboratory of Neuropsychophysiology, Faculty of Psychology and Education Sciences, University of Porto, Porto, Portugal; ^7^Department of Pharmacology and Therapeutics, Faculty of Veterinary Medicine, Damanhour University, Damanhour, Egypt

**Keywords:** COVID-19, SARS-CoV-2, diabetes mellitus, hyperglycemia, cardiometabolic disturbances

## Abstract

Coronavirus disease 2019 (COVID-19), triggered by the severe acute respiratory syndrome-coronavirus 2 (SARS-CoV-2), may lead to extrapulmonary manifestations like diabetes mellitus (DM) and hyperglycemia, both predicting a poor prognosis and an increased risk of death. SARS-CoV-2 infects the pancreas through angiotensin-converting enzyme 2 (ACE2), where it is highly expressed compared to other organs, leading to pancreatic damage with subsequent impairment of insulin secretion and development of hyperglycemia even in non-DM patients. Thus, this review aims to provide an overview of the potential link between COVID-19 and hyperglycemia as a risk factor for DM development in relation to DM pharmacotherapy. For that, a systematic search was done in the database of MEDLINE through Scopus, Web of Science, PubMed, Embase, China National Knowledge Infrastructure (CNKI), China Biology Medicine (CBM), and Wanfang Data. Data obtained underline that SARS-CoV-2 infection in DM patients is more severe and associated with poor clinical outcomes due to preexistence of comorbidities and inflammation disorders. SARS-CoV-2 infection impairs glucose homeostasis and metabolism in DM and non-DM patients due to cytokine storm (CS) development, downregulation of ACE2, and direct injury of pancreatic β-cells. Therefore, the potent anti-inflammatory effect of diabetic pharmacotherapies such as metformin, pioglitazone, sodium-glucose co-transporter-2 inhibitors (SGLT2Is), and dipeptidyl peptidase-4 (DPP4) inhibitors may mitigate COVID-19 severity. In addition, some antidiabetic agents and also insulin may reduce SARS-CoV-2 infectivity and severity through the modulation of the ACE2 receptor expression. The findings presented here illustrate that insulin therapy might seem as more appropriate than other anti-DM pharmacotherapies in the management of COVID-19 patients with DM due to low risk of uncontrolled hyperglycemia and diabetic ketoacidosis (DKA). From these findings, we could not give the final conclusion about the efficacy of diabetic pharmacotherapy in COVID-19; thus, clinical trial and prospective studies are warranted to confirm this finding and concern.

## Introduction

Novel coronavirus disease 2019 (nCov19) or coronavirus disease 2019 (COVID-19) is a recent viral infectious disease that emerged in December 2019; it was first identified in Wuhan, Hubei Province, China and presented as clusters of pneumonia, formally known as a pneumonia cluster of unknown etiology. COVID-19 is triggered by severe acute respiratory syndrome-coronavirus 2 (SARS-CoV-2) (1). Some years ago, namely, in 2003 and 2012, respectively, severe acute respiratory syndrome coronavirus (SARS-CoV) and Middle East respiratory syndrome coronavirus (MERS-CoV) led to fatal pneumonia, acute lung injury (ALI), and acute respiratory distress syndrome (ARDS). SARS-CoV, MERS-CoV, and SARS-CoV-2 are positive-sense, enveloped single-strand RNA *Betacoronaviruses* with some phylogenetic similarities. As a matter of fact, SARS-CoV-2 presents 79% similarity with SARS-CoV and 96% with bat CoV. In addition, it has been shown that SARS-CoV-2 has a higher ability of transmission and a lower fatality rate compared to SARS-CoV and MERS-CoV (2).

Declared on 30 January 2020 as a Public Health Emergency of International Concern by the World Health Organization (WHO), COVID-19 was quickly renamed to a pandemic on 11 March 2020, and on 3 November 2020, 47,705,405 confirmed cases were officially reported in more than 200 countries with 1,217,347 deaths globally (3).

The incubation period of SARS-CoV-2 is 2–14 days; however, some studies have reported that COVID-19 symptoms are developed in 97.5% of cases within 4–5 days. SARS-CoV-2 is mainly transmitted by respiratory droplets up to a distance of 6 ft. This virus remains viable for about 3 h in the aerosols and can be transmitted in closed environments. Nonetheless, viable SARS-CoV-2 has also been detected in fecal swabs; thereby, transmission of SARS-CoV-2 through the fecal-oral route might be an important route, mainly in patients on drugs that upregulate intestinal angiotensin receptor type 2 (ACE2) (4). The age group most affected by COVID-19 is mainly between 47 and 59 years, where men are more prone to the disease. Fewer COVID-19 cases have been reported in infants and children; as disclosed by a large cohort study in China, only 2% of COVID-19 cases were below the age of 20 (5). Regarding clinical presentation, the clinical spectrum of COVID-19 presents mainly as asymptomatic or mild flu-like illness in 85%, mostly in children and adults, although in 10% of cases, a severe disease state may be present, along with an increased risk of developing ARDS. In addition, in severe cases, COVID-19 may trigger extrapulmonary manifestations like acute cardiac injury, arrhythmias, acute kidney injury, acute brain injury, endocrine failure, multiple organ failure, and even death (6).

Diabetes mellitus (DM) is an endocrine disorder characterized by hyperglycemia, polyuria, polydipsia, and weight loss due to a defect in insulin secretion and/or action. DM is commonly associated with metabolic, macrovascular, and microvascular complications that increase morbidity and mortality in different viral infections (7). It has been reported that DM and reactive hyperglycemia are regarded as predictors of severity in SARS-CoV and MERS-CoV infected patients (8). However, little is known about the association between SARS-CoV-2 and DM; nevertheless, different recent studies observed the link between hyperglycemia and SARS-CoV-2 even in non-DM patients (9). Therefore, this review aims to provide an overview of the potential link between COVID-19 and hyperglycemia as a risk factor for DM development.

## Method and Search Strategy

In order to review the report of ethical considerations in these papers, we proposed a protocol for a systematic review of the COVID-19 articles. The search criteria proposed for the review were based on what would be a reasonable search conducted by a lay member of the public with access to PubMed.gov. It was proposed to publish the findings of the review as a summary of the institutional Research Ethics Committee's response to the challenges of reviewing and approving clinical research proposals in times of a pandemic.

To accomplish that, a systematic search in MEDLINE through Scopus, Web of Science, PubMed, Embase, China National Knowledge Infrastructure (CNKI), China Biology Medicine (CBM), and Wanfang Data was done using the following terms and keywords: [COVID-19] OR [SARS-CoV-2] OR [2019-nCov] OR [Wuhan virus] AND [DM] OR [hyperglycemia] OR [Pancreatic injury]. There were no limitations for language and type of published articles as well as preprinted data.

## COVID-19 and Hyperglycemia

It has been stated that COVID-19 is associated with hyperglycemia, actually considered a direct predictor of the poor prognosis of the disease and to an increased risk of death (10). Briefly, the binding site and entry point of SARS-CoV-2 is the ACE2 receptor, which is highly expressed in the lung, liver, brain, placenta, and pancreas. SARS-CoV-2 infects the pancreas through ACE2, being highly expressed there when compared to other organs, leading to pancreatic damage with subsequent impairment of insulin secretion and development of hyperglycemia even in non-DM patients. Similarly, SARS-CoV-2-induced pancreatic injury may worsen a preexistent DM (11). Previous data have shown that SARS-CoV, which is closely related to SARS-CoV-2, triggers transient hyperglycemia and impairment of pancreatic β-cell function during epidemic-derived pneumonia (12). Moreover, the COVID-19-induced inflammation and cytokine storm (CS), which are characterized by profound elevations in the levels of tumor necrosis factor-alpha (TNF-α) and interleukin (IL)-6, lead to peripheral insulin resistance (IR) (13). Besides, high TNF-α and IL-6 in CS impair pancreatic β-cell function and inhibit insulin secretion. Taken together, both IR and impairment of pancreatic β-cell function contribute to a vicious cycle in the development and progression of hyperglycemia in COVID-19 patients (14). Furthermore, hyperglycemia and induced oxidative stress and gluco-lipotoxicity contribute to the development of IR and impairment of pancreatic β-cell function (15). In addition, prolonged hyperglycemia could worsen the course of COVID-19 via glycation of pancreatic ACE2, which facilitates the SARS-CoV-2 binding and entry at the pancreatic β-cell (16).

Different reports have shown that an abnormal expression of cell ACE2 receptors in different tissues reduces the protective effect against viral entry and, consequently, exacerbates the severity and poor outcomes of SARS-CoV-2 infection (2). On the other hand, the systemic renin-angiotensin system (RAS) regulates pancreatic β-cell function, while local RAS of pancreatic β-cell function controls β-cell apoptosis, cell proliferation, and oxidative stress (17). Angiotensin II (AngII) through AT1R leads to DM induction in experimental models, while inhibiting the glucose-stimulated insulin secretion. Therefore, blockade of AT1R improves pancreatic β-cell function and increases the pro-insulin and insulin biosynthesis. Besides, upregulated local pancreatic AngII induces oxidative stress that triggers β-cell damage by NADPH oxidase induction (18). Indeed, it has been shown that hyperglycemia upregulates AT1R leading to β-cell function impairment and insulin secretion (19).

**Figure 1:**
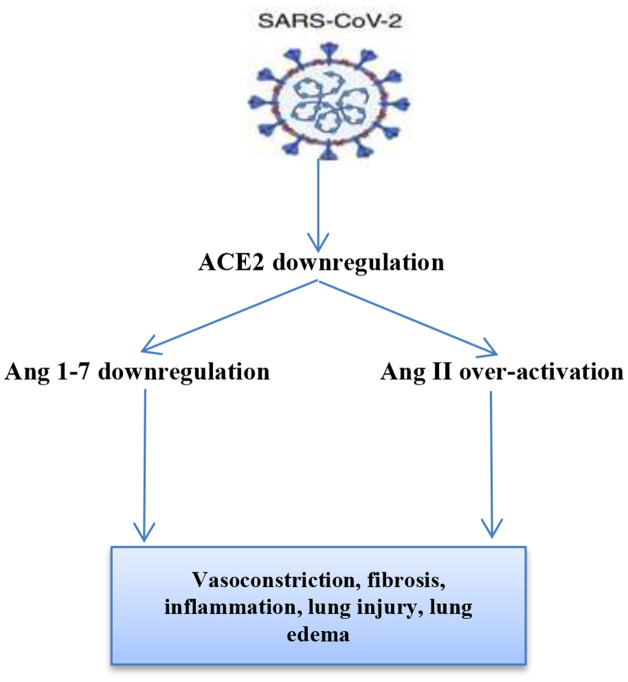
SARS-CoV-2 and renin-angiotensin system (RAS) interaction: SARS-CoV-2 down-regulates ACE2 leading to over-activation of AngII and reduction of Ang 1-7. Ang II through angiotensin receptor type 1 (AT1R) leads to lung injury, inflammation, and vasoconstriction. Ang 1-7 through Mas receptor leads to vasodilation and lung protection.

In COVID-19, ACE2 dysregulation by SARS-CoV-2 leads to marked elevation of vasoconstrictor AngII with a reduction in the vasodilator Ang1-7 (Figure 1), which *per se* leads to pancreatic β-cell dysfunction, inhibition of insulin secretion, and hyperglycemia, which might be transient even in non-DM patients (20). Furthermore, elevated AngII leads to pulmonary vasoconstriction, ALI, and ARDS with the induction of inflammation cascade and oxidative stress, which together participate in the induction of pancreatic β-cell function and hyperglycemia (21). Then, hyperglycemia in COVID-19 leads to ALI through the induction of pulmonary sodium-potassium-chloride co-transporter 1(NKCC1), involved in the regulation of the transport of water and ions to alveolar cells. Thus, untreated and long-standing hyperglycemia may lead to ALI through ischemic-reperfusion injury (22). Also noteworthy is the fact that hyperglycemia is associated with oxidative stress' induction and inflammatory mediators' overproduction, which together partake in the development of endothelial dysfunction and thrombosis due to alterations in both function and generation of antithrombin III (23). Taken together, these findings reveal that both COVID-19 and hyperglycemia interact in a vicious cycle leading to more complications and worse metabolic outcomes.

## COVID-19 and Pancreatic Injury

Pancreatic injury (PI) is often presented as acute pancreatitis, which is rarely reported in COVID-19 and may be misdiagnosed by the general signs and symptoms of acute viral infections (24). Different studies reported that COVID-19-induced PI is diagnosed through detailed medical history, physical examination, and ultrasonography imaging with elevation in serum lipase levels. However, in severe cases, abdominal CT scan imaging is recommended (25). In COVID-19, PI may occur either by direct invasion of SARS-CoV-2 or indirectly through the induction of CS (26). Previously, SARS-CoV was detected in the pancreatic tissue suggesting binding of this virus to ACE2, highly expressed in the pancreatic tissue mainly in β-cell and exocrine ducts (27). These findings suggest that the direct cytopathic effect of SARS-CoV-2 may be linked with the development of PI.

**Figure 2:**
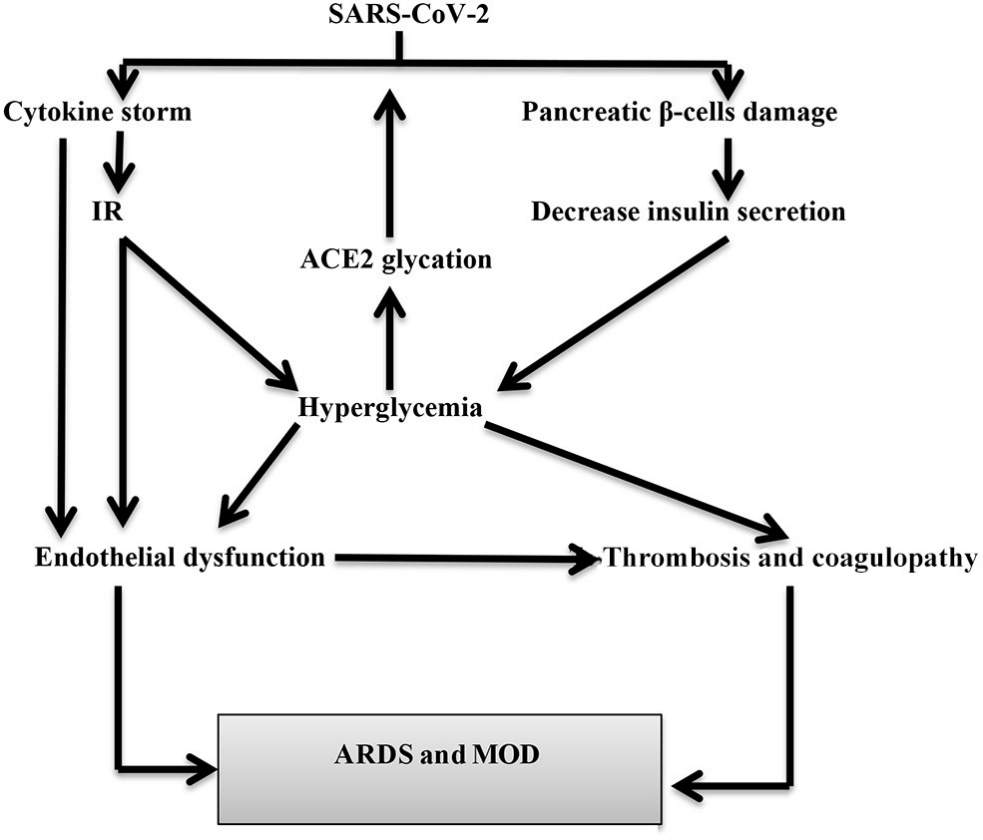
SARS-CoV-2-induced hyperglycemia in COVID-19 patients. SARS-CoV-2 leads to hyperglycemia either directly through pancreatic β-cells injury or indirectly through cytokine storm-induced insulin resistance (IR). Hyperglycemia increased SARS-CoV-2 entry through glycation of ACE2. Hyperglycemia may cause acute respiratory distress syndrome (ARDS) and multiorgan damage (MOD) through the induction of endothelial dysfunction and coagulopathy.

In SARS-CoV-2, the systemic inflammatory response and CS may be the cause of PI as part of multiorgan failure. SARS-CoV-2-induced PI increases the release of pancreatic lipase causing lipolysis and the release of unsaturated fatty acids that ultimately trigger pancreatic mitochondrial damage and overproduction of pro-inflammatory mediators similar to that of CS (24). Recent reports disclosed that SARS-CoV-2 affects both pancreatic lipase and peripheral adipose tissue leading to PI and lipotoxicity that contribute to CS induction (28). Also, postmortem studies in both SARS-CoV and SARS-CoV-2 patients illustrated a higher proliferation of these viruses in the pancreatic tissues (29). Therefore, SARS-CoV-2 may lead to PI directly or indirectly with subsequent endocrine and exocrine dysfunctions that are presented as acute pancreatitis and transient hyperglycemia (30, 31) (Figure 2).

Also, IL-6 is regarded as a potential link between ALI and PI in mice since PI-induced inflammations activate myeloid cells to secrete IL-6. In COVID-19-induced CS, IL-6 is the main cytokine involved in ALI and ARDS development (32). The binding of SARS-CoV-2 to the pancreatic β-cell ACE2 receptor stimulates A disintegrin and metalloprotease-17 (ADAM-17), which activate the ACE2 receptor shedding and TNF-α production (33). Therefore, SARS-CoV-2 infection is linked with the downregulation of ACE2 and the dysregulation of systemic and local pancreatic β-cell RAS. In this sense, the administration of recombinant soluble ACE2 neutralizes SARS-CoV-2 and prevents further viral entry with significant amelioration of pancreatic function (34).

**Figure 3:**
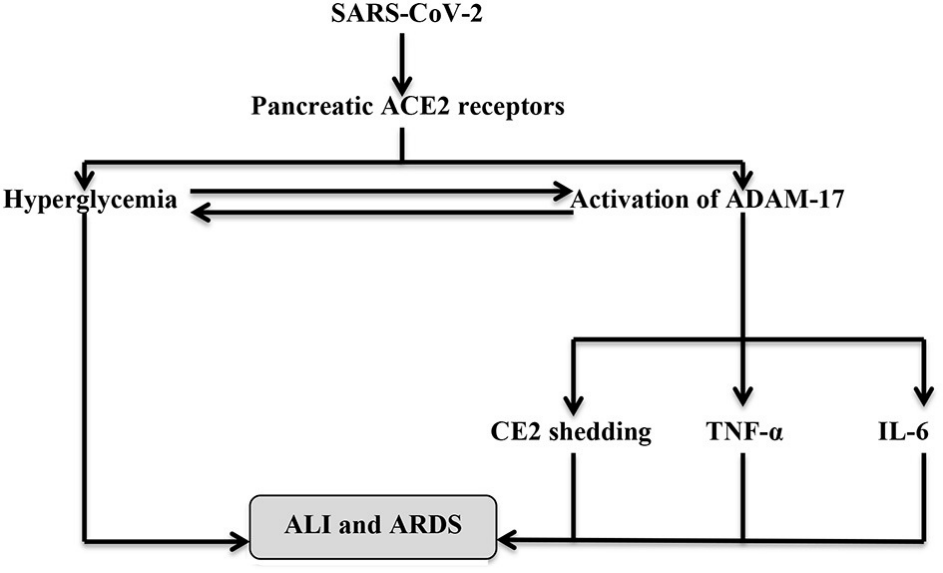
The interaction between SARS-CoV-2 and pancreatic ACE2. Binding of SARS-CoV-2 to the pancreatic ACE2 leads to pancreatic injury (PI)-induced hyperglycemia and activation of A disintegrin and metalloprotease-17 (ADAM-17), which activate shedding of ACE2 receptors and production of TNF-α and IL-6. Hyperglycemia activates ADAM-17 and vice versa. These changes together participate in the development of ALI and ARDS.

It has been shown that ACE2 deficiency alters glucose homeostasis and metabolism since ACE2 knockout mice illustrated AngII-independent pancreatic β-cells dysfunction, suggesting a direct protective role of the β-cell ACE2 receptor (35). Furthermore, overexpression of the β-cell ACE2 receptor may improve glucose homeostasis and β-cell sensitivity, while the downregulation of the peripheral ACE2 receptor is linked to the development of IR through the reduction of glucose transporter 4 (GLUT4) and Ang1-7, which increases peripheral insulin sensitivity (36). Liu et al. (37) illustrated that ACE2 polymorphism is associated with the development of type 2 DM. Also, ACE2 receptors have been reported to act as a compensatory mechanism against hyperglycemia induced-RAS activation since hyperglycemia activates ADAM-17 and ACE2 renal shedding that are common in patients with T2DM and IR. Also, ADAM-17 activation by SARS-CoV-2 leads to hyperglycemia. These changes augment the action of AngII on AT1R leading to vasoconstriction, hypertension, endothelial dysfunction, and hypercoagulation status (38). Previously, SARS-CoV infection was associated with 50% of acute DM cases due to a reduction in the pancreatic β-cell ACE2 receptors by direct viral invasion; nevertheless, only 10% of them developed chronic DM after 3 years (39). Besides, an augmented ADAM-17 during SARS-CoV-2 infection activates the release of pro-inflammatory cytokines, including IL-6 and TNF-α, which are correlated with a higher risk of ARDS and ALI (40). Hence, a rapid management of PI in COVID-19 patients may mitigate and attenuate the associated ALI. Therefore, the interaction between SARS-CoV-2 and pancreatic ACE2 not only causes PI but also may extend to cause systemic inflammatory changes (Figure 3).

## COVID-19 and Diabetes Mellitus

DM accounted for about 20% of the intensive care unit (ICU) admission due to COVID-19 according to cohort reports (41). However, data from Italy illustrated that more than two-thirds of COVID-19 patients who died had DM, with the mortality rate of DM patients with COVID-19 being similar to that of SARS and MERS (42).

During the previous SARS-CoV infection, non-DM patients may develop hyperglycemia on the 3rd day of acute infection that was reversed within 2 weeks (10% of them developed DM 3 years later). These finding are not observed in other viral pneumonia, suggesting the involvement of the pancreatic axis in coronavirus infection (43). In COVID-19, DM patients presented with preprandial and postprandial hyperglycemia as well as diabetic ketoacidosis, being higher compared to non-infected DM patients (44).

It has been accounted that any acute disease, as occurs in viral diseases, triggers stress and higher inflammatory responses that augment the sympathetic outflow with the release of catchecholamines, growth hormones, cortisol, and cytokines that together increase the frequency and severity of DM complications. However, in cases of coronavirus infection, the severity of such complications is also linked with the development of PI (45). Conversely, hypoglycemia may develop in SARS-CoV due to hepatic and pancreatic alpha cell injury, despite the fact that alpha cell dysfunction and lower glucagon serum levels were not confirmed in COVID-19 patients (46).

On the other hand, it was stated that DM increases the risk of COVID-19 progression and worsens the outcomes of other coronaviruses and H1N1 infections. The mortality rate of DM patients with COVID-19 is about 16% due to associated comorbidities and hidden presentation of mild disease. In fact, an underestimation of these signs and symptoms in DM patients may even worsen the outcomes in suspected SARS-CoV-2 infection (47).

Also, DM is linked to low-grade chronic inflammation, which may facilitate CS induced by COVID-19 pneumonia. The levels of IL-6, CRP, and D-dimer appear to be higher in COVID-19 pneumonia patients with DM. Of note, in DM patients with COVID-19, IL-6 links to associated metabolic disorders and cardiovascular complications, so IL-6 antagonist tocilizumab may attenuate the clinical course and outcomes in DM patients with COVID-19-induced pneumonia (48).

The interaction between DM and COVID-19 could be bi-directional, as SARS-CoV-2 infection may potentially deteriorate the preexisting DM and even predispose to frank DM in non-DM patients. In addition, pancreatic β-cell invasion by SARS-CoV-2 triggers β-cell autoimmunity in the susceptible subjects with subsequent development of type 1 DM (T1DM) (49).

The potential mechanisms that increase the risk of SARS-CoV-2 infection in DM patients are related to different metabolic pathways. It has been shown that DM patients with high body mass index, hypertension, and microvascular complications have a higher severity and mortality due to COVID-19-derived pneumonia (50). Also, hyperglycemia in DM individuals, independently, or secondarily to the presence of diabetic complications, increases the risk of SARS-CoV-2 infection in different ways, including an increased affinity from SARS-CoV-2 to ACE2, reduction of viral clearance, T cell-mediated immunity dysfunction, and CS induction (51).

In DM, there is a noteworthy disorder in the innate, adaptive, and acquired immunity with delay in the activation of Th1 cell-mediated immunity and late hyper-inflammation due to an abnormal cytokine response and alterations in CD4^+^ T cell counts. These abnormal immunological responses appear to be responsible for the blunted antiviral response in DM (52). Moreover, in DM, there is an overexpression of ACE2 in lung, kidney, heart, and pancreas that favors SARS-CoV-2 binding and entry (52, 53). Besides, the associated anti-DM pharmacotherapy and other administrated drugs in DM patients may affect the expression of ACE2 (53). However, insulin therapy reduces the expression of ACE2, while metformin, glucagon-like peptide-1 agonist, thiazolidinediones, statins, and ACE inhibitors upregulate the expressions of ACE2 (54).

**Figure 4:**
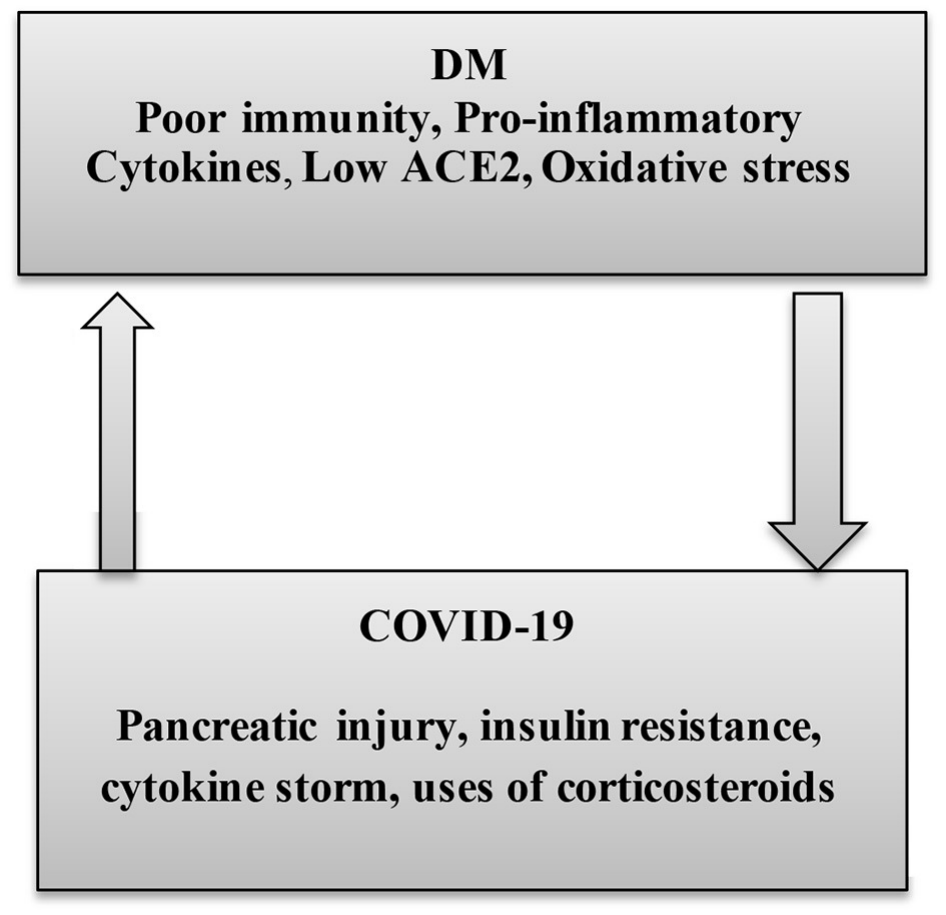
The interaction between diabetes mellitus and COVID-19. In diabetes mellitus (DM), poor immunity, high pro-inflammatory cytokine, low ACE2, and oxidative stress increase susceptibility for COVID-19. Below, COVID-19-induced pancreatic injury, insulin resistance (IR), cytokine storm, and uses of corticosteroids in the management of COVID-19 collectively participate in the induction of DM.

Nonetheless, the causal relationship between DM and ALI in COVID-19 cases, to what concerns to the expression of ACE2, is not yet clear. However, a recent study confirms that long-standing DM is linked to an overexpression of pulmonary ACE2 (55). Also, the susceptibility of DM patients to SARS-CoV-2 infection is also related to a higher furin serum level, which is engaged in the S-domain cleaving of spike protein and increasing the SARS-CoV-2 binding to the ACE2 receptors (56). Interestingly, the pulmonary ACE/ACE2 ratio is increased in DM, which favors the generation of vasoconstrictor AngII, involved in the induction of ALI. Also, in DM patients, high AngII levels and SARS-CoV-2 infection interact mutually at a vascular endothelial bed causing endothelial dysfunction, inhibition of fibrinolytic system, and activation of coagulation cascades that trigger thromboembolic disorders (57). Thus, there is a mutual interaction between DM and SARS-CoV-2 in COVID-19 (Figure 4).

## Anti-COVID-19 Medications and Blood Glucose Variability

Currently used drugs for COVID-19 treatment may affect blood glucose variability in both DM and non-DM patients. For example, chloroquine and hydroxychloroquine have been shown to be effective in controlling SARS-CoV-2 replications and in modulating COVID-19-induced CS due to their potent anti-inflammatory and immunomodulating effects (58). It has been reported that hydroxychloroquine improves glycemic indices, β-cell function, and insulin secretion and can be effectively used in the management of uncontrolled T2DM. Thus, hydroxychloroquine therapy in COVID-19 may lead to hypoglycemia since this drug reduces insulin degradation and improves insulin storage with augmentation of peripheral glucose metabolism (59). Therefore, cautions should be regarded in the use of hydroxychloroquine for treating COVID-19 patients with DM.

Corticosteroids, such as dexamethasone, are approved drugs since a long time ago that have shown to be effective in COVID-19 patients, namely, reducing the exaggerated immune response-induced ALI and ARDS. Despite this beneficial effect, dexamethasone blocks both viral clearance and immune response (60). In addition, administration of corticosteroids in COVID-19 patients is associated with hyperglycemia even in non-DM patients. In clinical practice, short-term therapy of low-dose methylprednisolone (30–80 mg/day for 3–5 days) is ineffective for COVID-19 management; however, a high-dose methylprednisolone (80–160 mg/day for 7 days) triggers an effective action in suppressing CS, and this high dose may aggravate hyperglycemia in DM (61, 62). Therefore, a strict glucose monitoring is crucial for COVID-19 patients who receive corticosteroids to prevent hyperglycemia-induced complications.

## Diabetic Pharmacotherapy and COVID-19

Diabetes pharmacotherapy may affect the clinical course and outcomes in DM patients with COVID-19 through modulation of ACE2 expression and potential anti-inflammatory effects.

### Metformin

Metformin improves IR and peripheral glucose uptake through the activation of AMP-dependent protein kinase. Also, metformin exerts pleiotropic effects through the AMP-independent pathway including anti-inflammatory and immunomodulatory effects (63); it inhibits the synthesis and release of CRP, IL-1β-induced IL-6, and ferritin from macrophages, endothelial cells, smooth muscle vascular cells, and hepatocytes (64). An observational study illustrated that metformin reduces the mortality rate in DM patients with severe COVID-19, more evident in women than men, through the suppression of TNF-α synthesis and release (65). It has been reported that up to 88% of T2DM patients receive metformin as first-line therapy, and since COVID-19 is common in DM, metformin may affect this pandemic (66). Specifically what concerns COVID-19 is that metformin upregulates ACE2 with increases in its stability and acts synergistically with ACEIs in the overexpression of pulmonary ACE2 receptors. As a consequence, the overexpression of pulmonary ACE2 receptors may attenuate the deleterious effect of SARS-CoV-2 invasion in alveolar cells while restoring the RAS balance (67). Metformin also reduces the binding of SARS-CoV-2 to ACE2 through inducing functional changes in the transmembrane enzyme by AMP-dependent phosphorylation (68). In addition, metformin blocks the mammalian target of rapamycin (mTOR) signaling, which is an important signaling pathway involved in viral pathogenesis and replication, such as influenza, SARS, MERS, and SARS-CoV-2; thus, metformin may attenuate viral replication through preventing the interaction of the viral protein complex (69). It has been documented that metformin therapy in DM patients inhibits the Zika virus replication through the activation of AMP signaling, which might be applied against SARS-CoV-2 (70). Add to this the fact that metformin therapy in DM patients with COVID-19 improves insulin sensitivity, and so it prevents the IR-induced overexpression of pancreatic ACE2. It is well-known that IR is linked to the development of cardiometabolic disorders that favor COVID-19 complications in DM (71).

**Figure 5:**
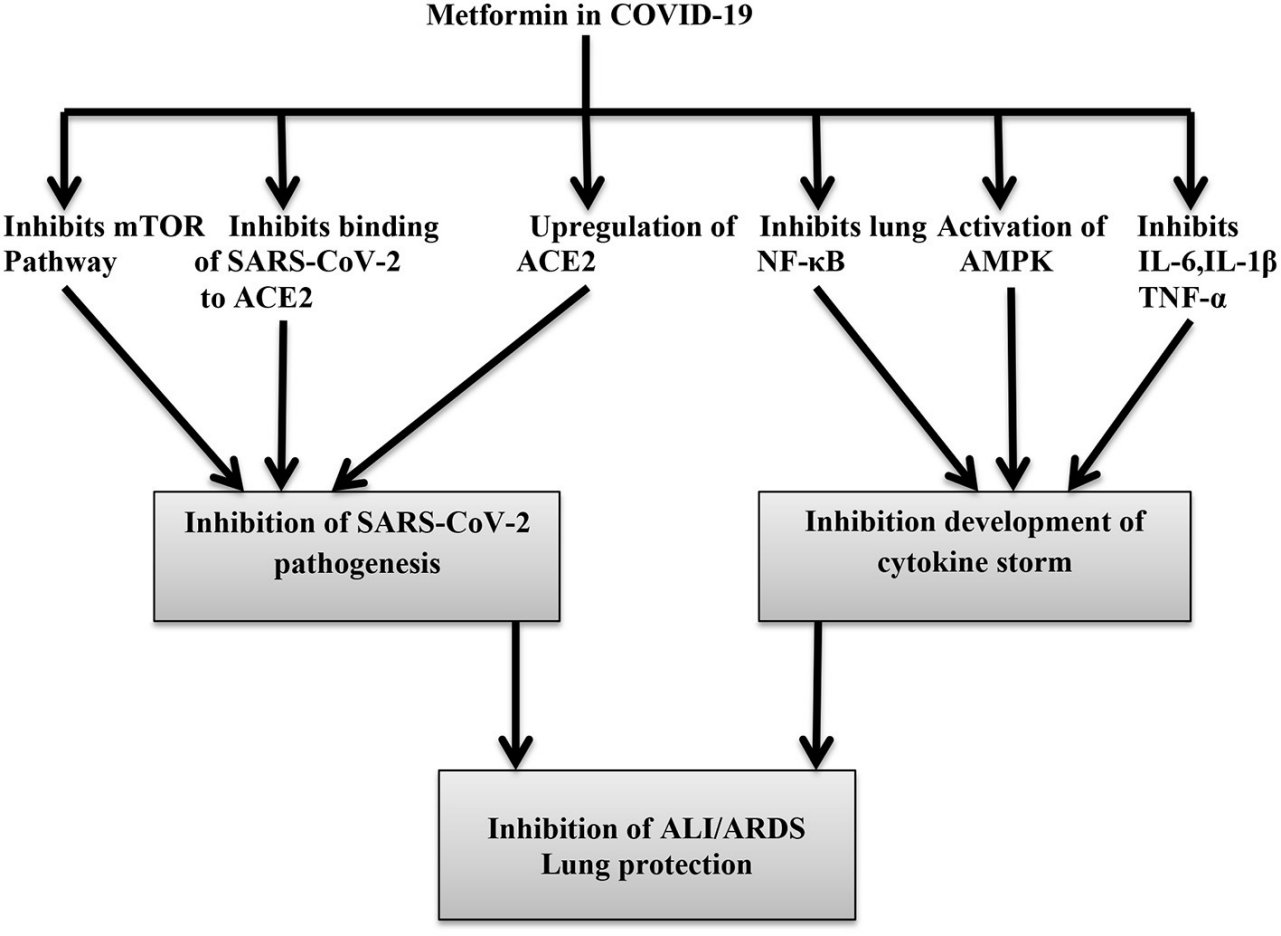
Metformin inhibits pathogenesis of COVID-19 through inhibition of pro-inflammatory cytokines, inflammatory signaling pathway, and binding to ACE2.

Different substantial data have shown that metformin leads to a decrease in the generation of reactive oxygen species (ROS) through the inhibition of the mitochondrial respiratory chain and 3-kinase phosphoinosis (PI3K)-Akt-dependent inflammatory response in lung tissue (71). Furthermore, it also inhibits nitric oxide (NO), prostaglandin E2 (PGE-2), and NF-kB in lung macrophages during SARS and MERS infection. However, administration of metformin in COVID-19 should be weighed against the risk of lactic acidosis and kidney impairment, which are commonly associated with COVID-19 pneumonia (72). Thus, regardless of its glucose-lowering abilities, metformin is recommended in COVID-19 pneumonia due to its potent anti-inflammatory effects. Al-kuraishy et al. (73) observed that metformin is effective in the reduction of COVID-19 severity and associated complications, such as ALI and acute ischemic stroke (AIS), through the modulation of SARS-CoV-2-induced inflammatory reactions in COVID-19 patients with T2DM. Nonetheless, its use is contraindicated in COVID-19 patients with lactic acidosis, multiorgan failure, severe gastrointestinal disorders, and hypoxia (74). The potential benefit of metformin therapy in DM patients with COVID-19 is illustrated in Figure 5.

### Thiazolidinediones

Thiazolidinediones, such as pioglitazone and rosiglitazone, are classes of antidiabetic drugs that act through the activation of peroxisome proliferators-activated receptor-gamma (PPAR-γ) leading to the reduction of IR, the suppression of lipolysis, and the activation of lipogenesis with improvement of insulin sensitivity (75). Exactly, pioglitazone improves the peripheral glucose uptake and increases the pancreatic β-cell sensitivity, while also exerting an important anti-inflammatory effect through the suppression of monocytes and IL-6 release. Besides, pioglitazone reduces serum ferritin, CRP, and other pro-inflammatory cytokines in T2DM, thus reducing the likelihood of CS when COVID-19 is developed (76). Pioglitazone also reduces the SARS-CoV-2-induced IR and hyperglycemia in non-DM patients via the attenuation of ACE2 glycation and the dysregulation of RAS. Therefore, pioglitazone and other thiazolidinediones may have potential roles in the management of COVID-19-related complications (77).

**Figure 6:**
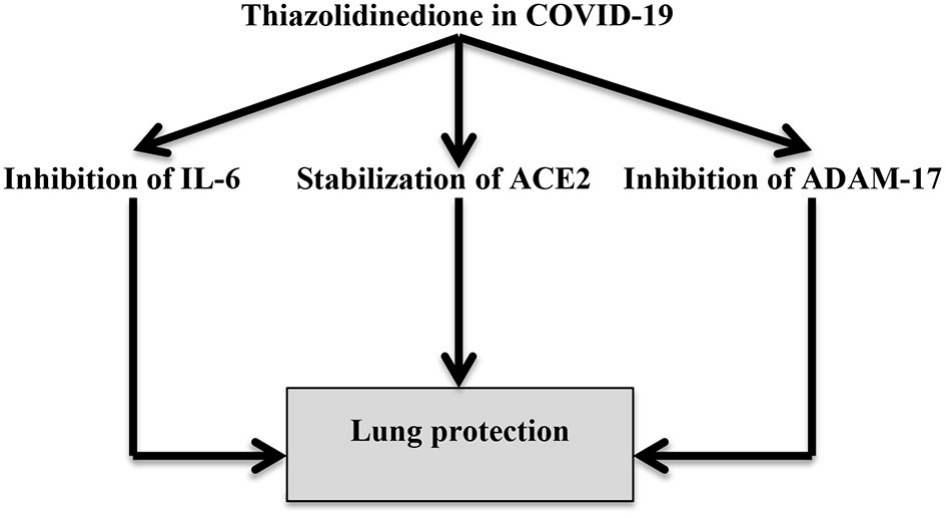
Thiazolidinedione attenuates the pathogenesis of COVID-19 through inhibition of IL-6 and ADAM-17 with the modulation of ACE2, as thiazolidinediones can regulate the AngII-mediated effects.

Moreover, thiazolidinediones attenuate pulmonary fibrosis and ALI by suppressing pulmonary myofibroblast differentiation and TGF-β signaling (78). For this reason, SARS-CoV-2 pathophysiology is related to its interaction with adipocytes and adipose-like cells that favor the differentiation of lung lipofibroblasts into myofibroblasts (79). Pioglitazone also increases ACE2 expression in insulin-sensitive tissues, normalizing blood glucose and attenuating acute kidney injury through the amelioration of the expression of renal ADAM17 (80). Therefore, thiazolidinediones can reduce the interaction between SARS-CoV-2 and adipocytes with subsequent reduction of COVID-19 severity (Figure 6).

### Dipeptidyl Peptidase-4 Inhibitors

Dipeptidyl peptidase-4 (DPP4) is a transmembrane glycoprotein type II expressed in different tissues and immune cells and plays an important role in the metabolism of glucagon-like peptide (GLP-1). DPP4 expression is higher in the visceral adipose tissue and involved in visceral inflammation and IR progression through enzymatic cleavage of cytokines and chemokines (81).

DPP4 inhibitors (DPP4Is) are oral hypoglycemic agents used in the management of T2DM acting through inhibiting the DPP4 enzyme, thereby increasing the incretin levels, which, in turn, increase insulin secretion with the reduction of glucagon secretion and blood glucose. Briefly, DPP4Is enhance the insulin secretion in a glucose-dependent manner (82).

Different studies have shown that DPP4Is exert anti-inflammatory and immunoregulatory effects in both autoimmune and inflammatory diseases (83). Among such drugs, sitagliptin, linagliptin, and vildagliptin reduce the CRP markers in T2DM patients within 12 weeks of treatment (76). However, there are no available data for other types of DPP4Is regarding their effects on CRP and ferritin serum levels in T2DM (84).

Concerning the viral infections, it has been confirmed that the DPP4 receptor is a recognized receptor for MERS-CoV that induces T-cell-dependent inflammatory reactions. So, antibodies directed against the DPP4 receptor inhibit MERS-CoV proliferation (85). In the context of the COVID-19 outbreak, DPP4Is and GLP-1 analog exert anti-adipogenic and anti-inflammatory effects that may reduce macrophage polarization and differentiation (86). Mirani et al. (51) showed that DM patients on DPP4I therapy developed a less severe pneumonia, with a lower need of mechanical ventilation and a lower mortality rate when developing COVID-19. For this reason, DPP4Is reduce COVID-19 virulence through the suppression of DPP4/CD26-dependent inflammatory signaling with subsequent inhibition of CS and disease progression. Recent evidences also suggest that SARS-CoV-2 interacts with both DPP4/CD26 and ACE2; besides, SARS-CoV-2 interacts with 293T-cells expressing DPP4 (87). Vankadari and Wilce (88) confirmed that sitagliptin triggers a marked inhibition of SARS-CoV-2 proliferation through binding to the F357 residue, causing conformational changes that prevent its binding with DPP4 receptors. These finding suggest that DPP4Is may attenuate SARS-CoV-2-induced ARDS by suppressing DPP4/CD26 signaling interactions. Therefore, the anti-inflammatory effects of DPP4Is may mitigate DM and coexisting COVID-19-induced IR, hyperglycemia, and inflammation (89).

**Figure 7:**
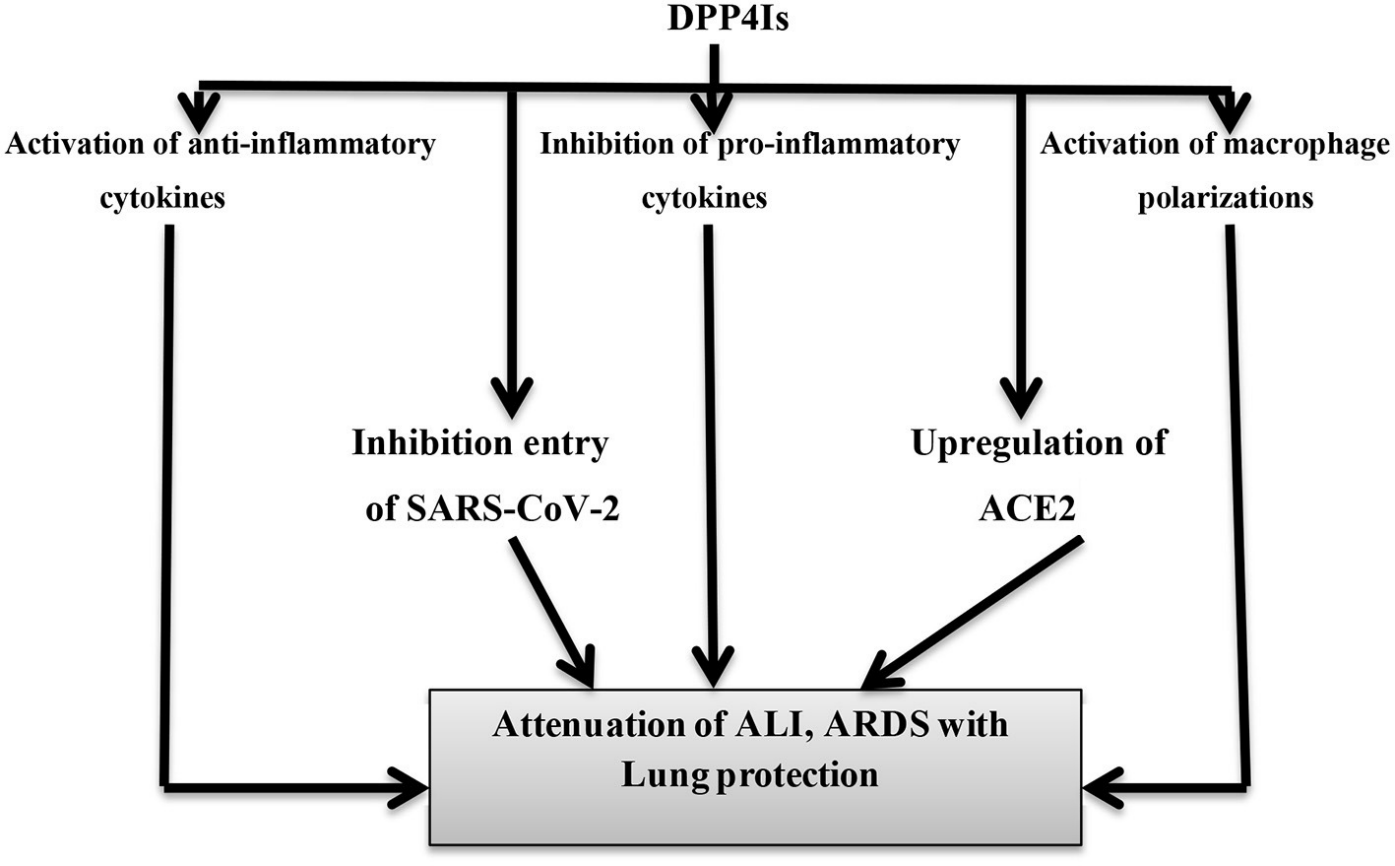
Potential effects of DPP4 inhibitors on the pathogenesis of COVID-19. Dipeptidyl peptidase-4 inhibitors (DPP4Is) have anti-inflammatory effects through the activation of anti-inflammatory cytokines, the activation of macrophage polarizations, and the inhibition of pro-inflammatory cytokines. DPP4Is inhibit the entry of SARS-CoV-2 through blocking of DPP4 receptors. The net effects of DPP4Is are lung protection with improvement in acute lung injury (ALI) and acute respiratory distress syndrome (ARDS).

On the other hand, the GLP-1 receptor analog (GLP-1RA), such as exenatide, has also potent anti-inflammatory and antiproliferative effects and plays a role in the attenuation of ALI. Exenatide improves the anti-inflammatory interleukin, IL-10, and inhibits pro-inflammatory cytokines, TNF-α and IL-1β, in the macrophage's/monocyte's axis (90). Also, the GLP-1 agonist, such as liraglutide, increases the ACE2 expression in the lungs and might have a protective role against the development of ALI in COVID-19-induced pneumonia (91). However, large prospective studies are recommended to confirm the potential role of DPP4Is and GLP-1RA in COVID-19. Therefore, DPP4Is have potential effects against SARS-CoV-2-induced ARDS through the modulation of anti-inflammatory and pro-inflammatory cytokines (Figure 7).

### Sodium-glucose Co-transporter-2 Inhibitors

Sodium-glucose co-transporter-2 inhibitors (SGLT2Is), also called gliflozins, which include canagliflozin, dapagliflozin, and empagliflozin, are a class of anti-DM drugs that inhibit SGLT2 at renal tubules and prevent glucose reabsorption (92). In addition, SGLT2Is reduce body weight and blood pressure, and exert anti-inflammatory effects through the reduction of IL-6, CRP, ferritin, and oxidative stress, thus being effective in mitigating ALI in T2DM (76). SGLT2Is play a role in COVID-19 management through the upregulation of protective ACE2, the attenuation of CS through the inhibition of IL-6 release, cytoprotective effect through the improvement of cell oxygenation, and reduction of lactate formation (93).

On the other hand, an elevated lactate level in COVID-19 reflects a status of hypoxia and anaerobic metabolism and is correlated with CS induction and multiorgan injury (94). Briefly, SARS-CoV-2 can induce anaerobic metabolism via the disruption of cell oxygenation and the induction of anaerobic glycolysis (95). As cell pH is controlled by Na^+^/H^+^ and lactate/H^+^ exchangers and symporters, respectively, high lactate serum levels in SARS-CoV-2 raise the activity of the lactate/H^+^ symporter with subsequent cell acidosis (96). Dapagliflozin inhibits cell Na^+^/H^+^ exchangers, thus reducing cell acidosis and SARS-CoV-2 activation at acidic pH. Similarly, other SGLT2Is also reduce lactate serum levels through the inhibition of lactate production and release, and increase urinary lactate excretion and LDH-dependent lactate formation (97). However, the risk of euglycemic DM ketoacidosis with SGLT2Is is very low (about 1%), but the risk of this side effect should be counterbalanced with the beneficial use of SGLT2Is in DM patients with COVID-19 (98). Hence, the net effect of SGLT2Is in COVID-19 is mainly related to the maintenance of cell pH with the reduction of the viral load. Into the bargain, SGLT2Is reduce IR and hyperglycemia-induced inflammatory reactions and ACE2 glycation, thereby reducing the risk of CS and AngII that are augmented in COVID-19 (99).

#### Sulfonylureas

Sulfonylureas (SU), such as glibenclamide, glipizide, and glimepiride, are a class of anti-DM agents that increase insulin secretion from pancreatic β-cells (100). SU has potent anti-inflammatory effects through the inhibition of IL-1β and nod-like receptor pyrine 3 NLRP3 inflammasome with antiplatelet effects and the reduction of thromboxane A2 activation (101). Platelets' aggregation and activation of both IL-1β and inflammasome are involved in CS generation in COVID-19 patients (102). SU blocks NLRP3 inflammasome-induced ALI through the suppression of the K^+^-ATP channel, K^+^ outflow block, the inhibition of Ca^2+^ entry and of oxidative stress, and the improvement of endogenous antioxidant capacity (103). However, the use of glibenclamide in DM patients with COVID-19 has not been evaluated since most DM patients with COVID-19 are switched to insulin therapy (104). Nonetheless, glibenclamide therapy in T2DM patients increases the risk of hypoglycemia, which might occur in COVID-19 patients (105, 106).

#### Insulin

Recent data have shown that insulin requirements are increased and correlated with high CRP serum levels and COVID-19 severity (107). For this reason, COVID-19-induced hyperglycemia, IR, and associated inflammatory disorders can increase the pancreatic β-cell burden in DM and non-DM patients (19). However, at the ICU, insulin requirements are higher in DM patients compared to controls due to the preexistence of an inflammatory status and cardiometabolic comorbidities (108). In COVID-19 patients, the direct interaction with pancreatic β-cells by SARS-CoV-2 leads to a significant reduction in insulin release. However, C-peptide is raised in COVID-19, suggesting that SARS-CoV-2 may cause transient pancreatic β-cell toxicity (109).

Thus, as hyperglycemia is commonly reported in critical illness, to ensure a proper control of hyperglycemia through insulin therapy, it is crucial to prevent the occurrence of cardiometabolic complications (110); therefore, early insulin therapy in critical illnesses, as occur in cases of COVID-19-induced hyperglycemia, may improve the clinical outcomes while reducing the mortality rate through different ways: (a) insulin inhibits pro-inflammatory cytokine linked to ARDS (111); (b) insulin promotes a restoration of pancreatic and renal ACE2 and ADAM-17 activity, and RAS balance (112); (c) insulin therapy reduces the risk of hyperglycemia and DKA that are associated with high mortality rates in COVID-19 patients. In addition, insulin therapy exerts a protective role against SARS-CoV-2-induced ALI and ARDS (113). To this effect, any COVID-19 patient should be monitored for blood glucose and HbA1c, along with a strict blood glucose monitoring, where insulin therapy should be properly administered. Besides, long-term evaluation of pancreatic β-cell function is recommended to ascertain potential β-cell damage and future DM development.

Moreover, higher CRP serum levels and neutrophil count reveal a humoral immune response in DM patients with COVID-19 (114). In fact, hyperglycemia affects antibody response during viral infection through the impairment of lymphocytes, macrophages, and neutrophil functions, as well as complement response (115). Therefore, the antibody response for SARS-CoV-2 vaccine may be impaired in DM due to hyperglycemia and IR (116). For these reasons, a strict insulin therapy is advisable to control the cell and humoral immune impairments in DM patients with COVID-19.

## Conclusions

Data obtained underlined that SARS-CoV-2 infection in DM patients is more severe and associated with poor clinical outcomes due to preexistent comorbidities and pro-inflammatory phenotype. SARS-CoV-2 infection impairs glucose homeostasis and metabolism in DM and non-DM patients due to cytokine storm (CS) development, downregulation of ACE2, and direct injury of pancreatic β-cells. Therefore, the potent anti-inflammatory effect of diabetic pharmacotherapies such as metformin, pioglitazone, sodium-glucose co-transporter-2 inhibitors (SGLT2Is), and dipeptidyl peptidase-4 (DPP4) inhibitors may mitigate the COVID-19 severity. In addition, some antidiabetic agents, including insulin, can reduce the SARS-CoV-2 infectivity and severity by modulating the expression of ACE2 receptors. Taken together, the data presented here illustrate that insulin therapy may seem as more appropriate than other anti-DM pharmacotherapies in the management of COVID-19 patients with DM due to the lower risk of uncontrolled hyperglycemia and reduced propensity to develop diabetic ketoacidosis (DKA). However, based on these findings, it is not yet possible to conclude decisively on the efficacy of diabetic pharmacotherapy in COVID-19, so thorough clinical trials are warranted.

## Author Contributions

All authors listed have made a substantial, direct and intellectual contribution to the work, and approved it for publication.

## Conflict of Interest

The authors declare that the research was conducted in the absence of any commercial or financial relationships that could be construed as a potential conflict of interest.
